# Gene expression profiles responses to aphid feeding in chrysanthemum (*Chrysanthemum morifolium*)

**DOI:** 10.1186/1471-2164-15-1050

**Published:** 2014-12-02

**Authors:** Xiaolong Xia, Yafeng Shao, Jiafu Jiang, Liping Ren, Fadi Chen, Weimin Fang, Zhiyong Guan, Sumei Chen

**Affiliations:** College of Horticulture, Nanjing Agricultural University, No.1 Weigang, Nanjing, 210095 China

**Keywords:** Chrysanthemum, Aphid, Gene expression, RNA-Seq

## Abstract

**Background:**

Chrysanthemum is an important ornamental plant all over the world. It is easily attacked by aphid, *Macrosiphoniella sanbourni*. The molecular mechanisms of plant defense responses to aphid are only partially understood. Here, we investigate the gene expression changes in response to aphid feeding in chrysanthemum leaf by RNA-Seq technology.

**Results:**

Three libraries were generated from pooled leaf tissues of *Chrysanthemum morifolium* ‘nannongxunzhang’ that were collected at different time points with (Y) or without (CK) aphid infestations and mock puncture treatment (Z), and sequenced using an Illumina HiSeq^TM^ 2000 platform. A total of 7,363,292, 7,215,860 and 7,319,841 clean reads were obtained in library CK, Y and Z, respectively. The proportion of clean reads was >97.29% in each library. Approximately 76.35% of the clean reads were mapped to a reference gene database including all known chrysanthemum unigene sequences. 1,157, 527 and 340 differentially expressed genes (DEGs) were identified in the comparison of CK-VS-Y, CK-VS-Z and Z-VS-Y, respectively. These DEGs were involved in phytohormone signaling, cell wall biosynthesis, photosynthesis, reactive oxygen species (ROS) pathway and transcription factor regulatory networks, and so on.

**Conclusions:**

Changes in gene expression induced by aphid feeding are shown to be multifaceted. There are various forms of crosstalk between different pathways those genes belonging to, which would allow plants to fine-tune its defense responses.

**Electronic supplementary material:**

The online version of this article (doi:10.1186/1471-2164-15-1050) contains supplementary material, which is available to authorized users.

## Background

Chrysanthemum (*Chrysanthemum morifolium* Ramat.) is an important ornamental plants with high economic value all around the world [1, 2]. It is susceptible to the aphid (*Macrosiphoniella sanbourni*) infestation from vegetative to flowering stage. *M. sanbourni* not only hampers the vegetative growth, but also decreases the quality of flowers, which causes serious loss in chrysanthemum production. Besides draining plants’ nutrients, aphids also transmit pathogenic viruses. The progress has been made in our understanding of plant-aphid interaction, especially the molecular bases of plant resistance and defense against aphid feeding. Several plant *R* (resistance) genes related with plant resistance to aphids have been identified. For example, *Mi-1.2* gene confers resistance to the potato aphid (*Macrosiphum euphorbiae* Thomas) in wild tomato, *Lycopersicon peruvianum* (L.) P. Mill. [3, 4]. And *Vat* (virus aphid transmission) gene mediates resistance to the cotton aphid (*Aphis gossypii* Glover) as well as some viruses transmitted by this aphid in melon, *Cucumis melo* L. [5]. Both *Mi-1.2* and *Vat* gene belong to the nucleotide-binding-site leucine-rich-repeat (NBS-LRR) family of plant *R* gene, and there is close linkage between resistance loci and NBS-LRR gene sequences revealed by genetic analysis [6, 7]. However, detailed mechanisms of *R* genes involved in aphid resistance still need further investigation.

During aphid infestation, series of plant defense responses, including plant hormone signal transduction, transcriptional regulation and the expression of defensive genes, would be induced [8]. Salicylic acid (SA), jasmonic acid (JA) and ethylene (ET) are three better studied phytohormones involved in aphid-induced plant hormone signal transduction. In interactions between *Myzus persicae* and *Arabidopsis thaliana*, SA signaling pathway is activated and the expression of genes such as pathogenesis-related (PR) genes (i.e., β-1,3-glucanase and chitinases) associated with the signaling pathway increased [9]. And a wide range of defensive responses in *Arabidopsis thaliana* attacked by cabbage aphid (*Brevicoryne brassicae*) depended on SA signaling [10]. Furthermore, recent studies found that SA signaling pathway was critical for *Mi-1.2*-mediated resistance to aphid [11]. The JA pathway, containing wound hormone JA-Ile, is also an important regulator of plant resistance to herbivores. Kusnierczyk *et al.* [12] indicated that many of defense-associated responses induced by cabbage aphid (*Brevicoryne brassicae*) in wild-type (WT) plants were impaired in *Arabidopsis* lacking jasmonates. JA promoted the synthesis of glucosinolate and N^δ^-acetyl ornithine in *Arabidopsis*, thereby improving the resistance to aphid [13, 14]. The resistance of alfalfa to blue green aphid (BGA) and defense of *Arabidopsis* on cabbage aphid are all dependent on the JA signaling cascades [10, 15]. Argandona and co-workers [16] observed that aphid feeding significantly induced the production of ET in the leaf tissue of aphid-resistant barley cultivars contrasted to susceptible ones. ET excited by green peach aphid infestation induced the expression of *AtMYB44*, which then bound to the promoter of *ETHYLENE INSENSITIVE2 (EIN2*), regulating the defense responses in *Arabidopsis* [17]. JA and ET often act synergistically, and are frequently antagonized by SA [18]. *EIN2* is a bifunctional transducer of ET and JA signal transduction [8]. Still, knowledge about members of the *EIN2* downstream defense pathway is limited.

The development of high-throughput technologies allows us a global view of gene expression changes during plant interactions with aphids. Moran *et al.* [19] suggested that genes associated with signaling, pathogenesis-related responses, oxidative stress and calcium-dependent signaling are crucial components of the aphid response profile in *A. thaliana*. Transcriptome and metabolome changes of *Arabidopsis* were investigated at 6, 12, 24 and 48 h after *B. brassicae* infestation to monitor the progress of early response by full-genome oligonucleotide microarrays, revealing reactive oxygen species (ROS) and calcium is involved in early signaling, JA and SA in the regulation of defense responses, and the induction of transcripts associated with senescence, biosynthesis of indolyl glucosinolates (IGS), anti-insect proteins, camalexin, and several WRKY transcription factors were identified as well [10]. Kusnierczyk and co-workers [12] conducted an extensive analysis of transcriptional patterns of WT, *aos* defective in JA production, and *fou2* constitutively inducing JA biosynthesis in *Arabidopsis*. More than 200 genes whose expression were dependent on jasmonate levels and over 800 genes that differentially responded to aphid feeding in *aos* and *fou2* plants than in WT were identified through microarray. They also demonstrated activation of defense caused by JA, such as *WRKY*, *ethylene responsive transcription factors* (*ERFs*), *BTB and TAZ domain protein 5* (*BT5*), *pathogenesis related proteins PR1* and *PR2*, and *plant defensines* (*PDFs*). Numerous key genes and proteins were unravelled in researches of gene transcriptional responses in model plants, such as *A. thaliana*, *Medicago truncatula*, *Nicotiana attenuata* and *Sorghum bicolor*. However, the exact mechanisms and functions of most of them are still unclear.

Previous studies on chrysanthemum found that superoxide dismutase (SOD), peroxidase (POD), ascorbate peroxidase (APX), polyphenol oxidase (PPO) activity and phenylalanine ammonia lyase (PAL) activities were enhanced by aphid herbivory, and changes in the enzymes activities in resistant species were faster than those in susceptible ones [20]. SA and MeJA pretreatment improved the resistance of chrysanthemum against aphids and increased the content of ROS species, defensive substances, flavonoids and lignins (data not shown), which implied that multiple pathways should be involved in the response of chrysanthemum to the aphid infestation. Therefore, to make a comprehensive view of differentially expressed genes (DEGs) during chrysanthemum-*Macrosiphoniella sanbourni* interaction, an experiment exploring comparative expression profiling was conducted. We also conduct a mock puncture treatment which is designed to partially simulate the mechanical stress resulting from aphid penetration, attempting to figure out the potential impacts of aphid stylets. This work would lay a foundation for further study in the resistance of chrysanthemum to aphid.

## Results

### An overview of three libraries data sets by RNA-Seq

Three libraries were generated from pooled leaf tissues of *Chrysanthemum morifolium* ‘nannongxunzhang’ that were collected at different time points with (Y) or without (CK) aphid infestations and mock puncture treatment (Z), and sequenced using an Illumina HiSeq^TM^ 2000 platform. After removing reads containing adaptor sequence and with low-quality, a total of 7,363,292, 7,215,860 and 7,319,841 clean reads were obtained, in library CK, Y and Z, respectively, corresponding to 360,801,308, 353,577,140 and 358,672,209 base pairs (Table 1) (Accession number SRS619289 for library CK; Accession number SRS627943 for library Y; Accession number SRS627944 for library Z). The proportion of clean reads was >97.29% in each library (Additional file 1: Figure S1). And these clean reads were deposited in the NCBI Sequence Read Archive database (http://trace.ncbi.nlm.nih.gov/Traces/sra_sub/sub.cgi?) under accession number SRP042216.

**Table 1:** **An overview of read mapping**

Sample ID	Clean reads	Total base pairs	Total mapped reads	Perfect match	<=2 bp mismatch	Unique match	Multi-position match	Total unmapped reads
CK	7,363,292 (100.00%)	360,801,308 (100.00%)	5,678,491 (77.12%)	3,591,545 (48.78%)	2,086,946 (28.34%)	3,743,517 (50.84%)	1,934,974 (26.28%)	1,684,801 (22.88%)
Y	7,215,860 (100.00%)	353,577,140 (100.00%)	5,444,023 (75.45%)	3,385,348 (46.92%)	2,058,675 (28.53%)	3,632,669 (50.34%)	1,811,354 (25.10%)	1,771,837 (24.55%)
Z	7,319,841 (100.00%)	358,672,209 (100.00%)	5,598,453 (76.48%)	3,510,566 (47.96%)	2,087,887 (28.52%)	3,699,741 (50.54%)	1,898,712 (25.94%)	1,721,388 (23.52%)

CK: control; Y: aphid infestation treatment; Z: mock puncture treatment.

**Figure 1:**
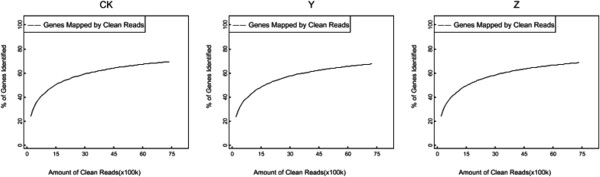
**Sequencing saturation analysis in the three libraries of CK, Y and Z.** CK: control; Y: aphid infestation treatment; Z: mock puncture treatment. The number of new detected genes rose as the read number was increased till above 6,000,000.

**Figure 2:**
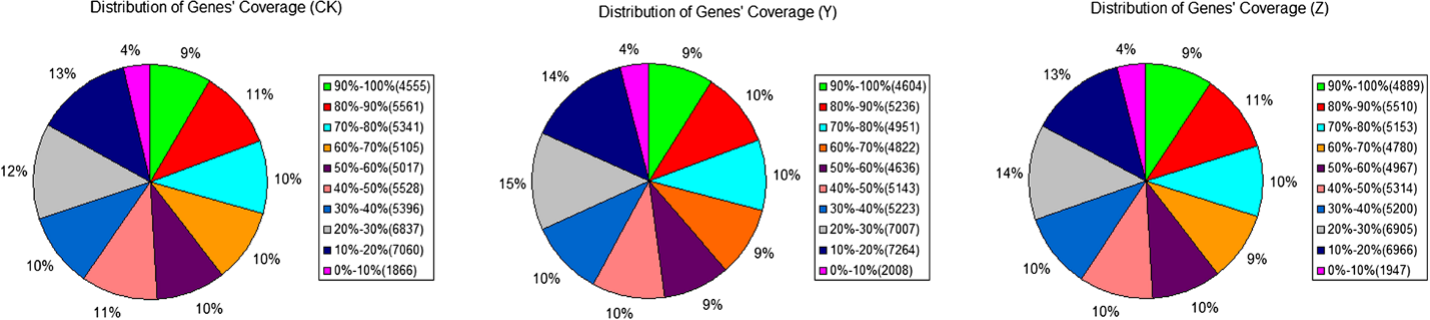
**Distribution of gene coverage in each library (CK, Y and Z).** CK: control; Y: aphid infestation treatment; Z: mock puncture treatment. The term “gene coverage” reflects the proportion of the full gene sequence represented by RNA-Seq reads.

A reference gene database including all known *Chrysanthemum morifolium* unigene sequences was applied to map the clean reads. According to the chosen criteria, an average of 76.35% of the clean reads were mapped (Table 1), which consisted of perfect match and < =2 bp mismatch. Regarding each library, the scales of clean reads uniquely mapped to the database were 50.84%, 50.34% and 50.54%, respectively. There were still approximately 23.65% of clean reads that cannot be mapped, mainly due to the restriction of the reference gene database of chrysanthemum. The number of genes identified increased with the number of reads until above 6,000,000, implying saturation of sequencing (Figure 1). The unigene coverage analysed as a means of evaluating the quality of the RNA-Seq data was mostly >50% (Figure 2).

### Differential expression and Gene ontology (GO) functional classification

**Figure 3:**
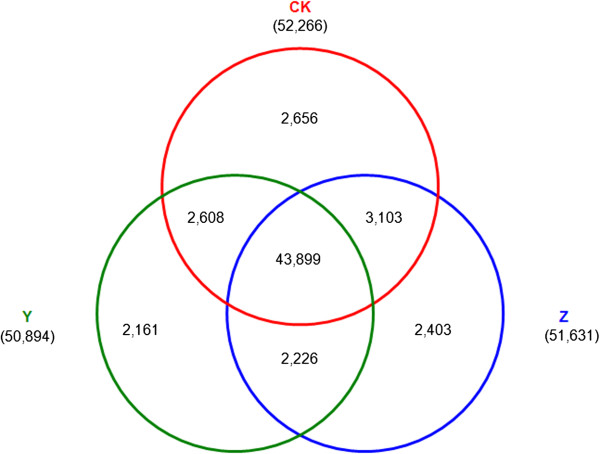
**The number of genes detected in library CK, Y and Z.** CK: control; Y: aphid infestation treatment; Z: mock puncture treatment.

In library CK, there are 52,266 genes detected, and 50,894 and 51,631 genes in library Y and Z, respectively. Among them, 2,656, 2,161 and 2,403 genes were specifically expressed in library CK, Y and Z, respectively; 46,507, 46,125 and 47,002 genes were co-expressed in library CK and Y, library Y and Z or library CK and Z, respectively; and 43,899 genes were simultaneously expressed in library CK, Y and Z (Figure 3).

**Figure 4:**
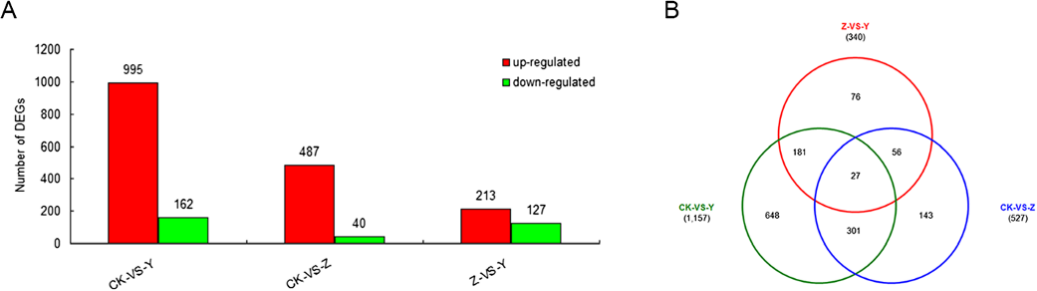
**The number of differentially expressed genes (DEGs) identified in CK-VS-Y, CK-VS-Z and Z-VS-Y comparison.** CK: control; Y: aphid infestation treatment; Z: mock puncture treatment. CK-VS-Y: comparison between CK and Y. CK-VS-Z: comparison between CK and Z. Z-VS-Y: comparison between Z and Y. The criteria used for assigning significance were: *P*-value < 0.05, FDR ≤ 0.001, and estimated absolute |log_2_Ratio(Y/CK)| ≥ 1. **A**: number of DEGs up- or down-regulated in CK-VS-Y, CK-VS-Z and Z-VS-Y comparison; **B**: number of DEGs specifically or co-expressed in CK-VS-Y, CK-VS-Z and Z-VS-Y comparison.

The transcript abundance of each gene was estimated by reads per kb per million reads (RPKM). Differentially expressed genes (DEGs) (Additional file 2: Table S1, Additional file 3: Table S2 and Additional file 4: Table S3) were identified according to Audic *et al.* [21], briefly *P*-value < 0.05, FDR ≤ 0.001, and estimated absolute |log_2_Ratio(Z/CK)| ≥ 1. Comparing the library CK with the library Y (CK-VS-Y), there were 1157 DEGs (995 genes up-regulated and 162 genes down-regulated, 995/162), and 527 (487/40) and 340 (213/127) DEGs in CK-VS-Z and Z-VS-Y, respectively (Figure 4A), of which 648, 143 and 76 genes were specifically expressed in CK-VS-Y, CK-VS-Z and Z-VS-Y, respectively; 328, 83 and 208 genes were co-expressed in CK-VS-Y and CK-VS-Z, CK-VS-Z and Z-VS-Y or CK-VS-Y and Z-VS-Y, respectively; and 27 genes were simultaneously expressed in CK-VS-Y, CK-VS-Z and Z-VS-Y (Figure 4B).

**Figure 5:**
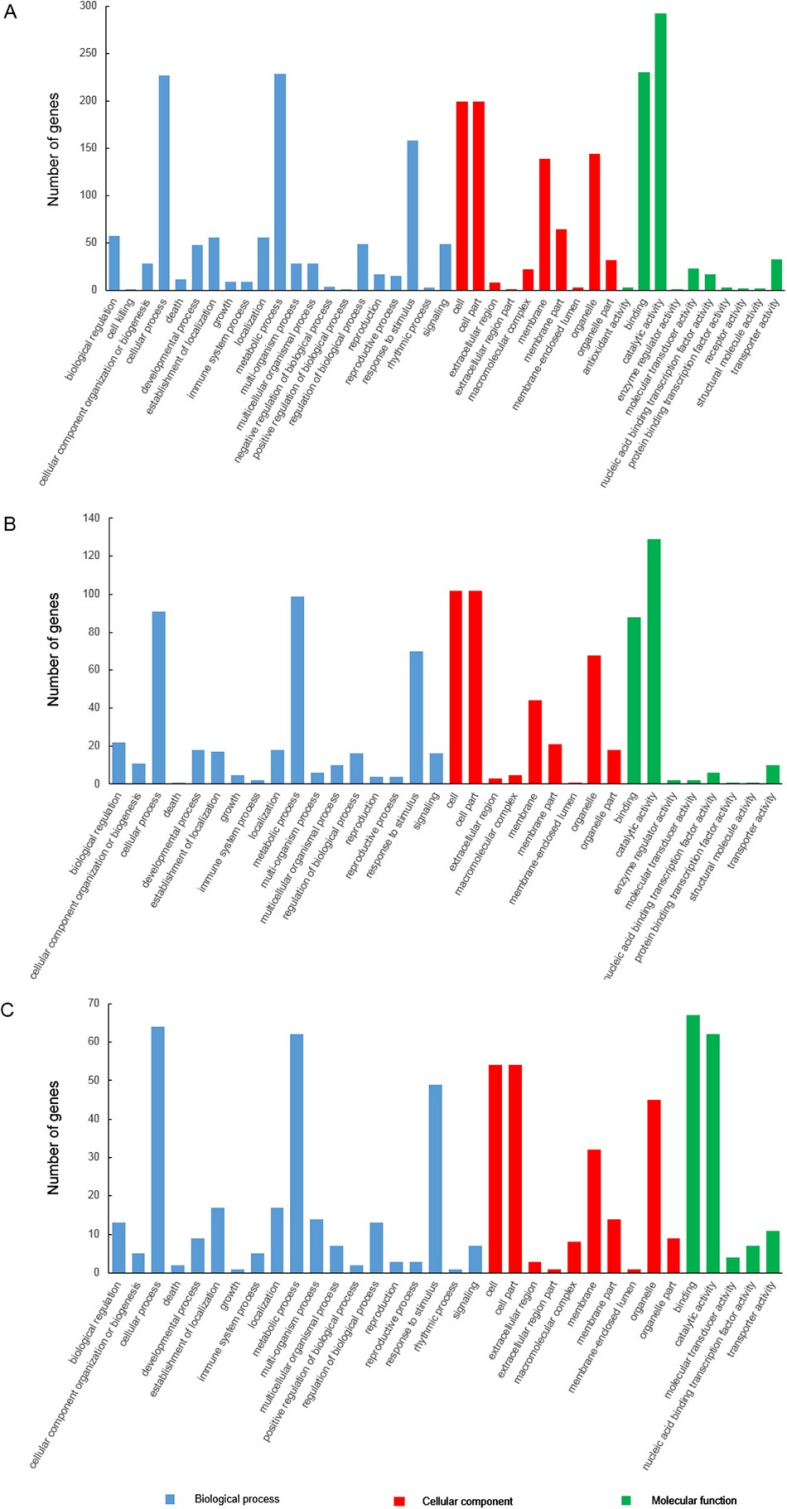
**Gene Ontology (GO) functional classification of differentially expressed genes (DEGs).** DEGs were annotated in three categories: biological process (blue), cellular component (red) and molecular function (green). CK: control; Y: aphid infestation treatment; Z: mock puncture treatment. **A**: comparison between library CK and Y (CK-VS-Y); **B**: comparison between library CK and Z (CK-VS-Z); **C**: comparison between library Z and Y (Z-VS-Y).

For CK-VS-Y, 477 out of 1157 DEGs (477/1157) could be assigned a GO classification, and 210/527 and 134/340 for CK-VS-Z and Z-VS-Y, respectively (Additional file 5: Table S4, Additional file 6: Table S5 and Additional file 7: Table S6). In CK-VS-Y comparison, 336 DEGs were categorized as “biological process”, 278 as “cellular component” and 378 as “molecular function” (336/278/378), and 136/121/159 and 97/72/100 in CK-VS-Z and Z-VS-Y, respectively (Figure 5). The number of DEGs in most categories and categories of DEGs in the CK-VS-Y was higher than in the CK-VS-Z and Z-VS-Y, such as ‘cell killing’, ‘regulation of biological process’, ‘response to stimulus’, ‘signaling’, and so on. Furthermore, in the CK-VS-Y (Figure 5A), most of DEGs were associated with cellular process, metabolic process and response to stimulus in terms of biological process, and in terms of cellular component, the majority were associated with cell, cell part, membrane and organelle, moreover, most were associated with binding and catalytic activity in terms of molecular function.

### Differentially expressed genes (DEGs) identified from aphid feeding and mock puncture treatments

In this study, hundreds of DEGs involved in different pathways in response to aphid feeding (CK-VS-Y) and mock puncture treatments (CK-VS-Z) were recognized. Some of them responded to both aphid infestation and puncture treatments, such as *NPR1*, *JAZ*, *MYC2* and *DELLA* involved in SA-JA-ET signaling network (Tables 2 and 3); *WRKY*, *MYB* and *AP2/ERF* transcription factors (Tables 4 and 5); ROS scavenging enzymes (Additional file 8: Table S7 and Additional file 9: Table S8); *COBRA-like* and *cellulose synthase like* (*Csl*) genes participating in cell wall biosynthesis (Additional file 10: Table S9 and Additional file 11: Table S10); and terpene synthase encoding genes in secondary metabolism (Additional file 12: Table S11 and Additional file 13: Table S12). Whereas, several DEGs were specifically expressed in aphid treatment, such as NADPH oxidase genes (Additional file 8: Table S7) and photosynthesis-related genes (Additional file 14: Table S13). Furthermore, individual members of a homologous gene family co-responding to aphid feeding and mock puncture treatment expressed preferentially either in CK-VS-Y or CK-VS-Z (Tables 2, 3, 4 and 5).

**Table 2:** **Differentially expressed genes (DEGs) involved in phytohormone metabolism and signaling pathway in the comparison between CK and Y (CK-VS-Y)**

GeneID	CK-RPKM	Y-RPKM	log _2_Ratio (Y/CK)	Up-Down-Regulation (Y/CK)	P-value	FDR	Gene description
Unigene107_All	6.80	21.82	1.68	up	6.52E-15	4.23E-13	NPR1-1 protein
Unigene23699_All	23.61	65.91	1.48	up	1.10E-21	1.08E-19	NIM1-like protein 1
Unigene16290_All	28.91	58.50	1.02	up	2.30E-05	0.000575	NIM1-like protein 1
Unigene2058_All	4.07	13.85	1.77	up	4.50E-07	1.47E-05	TGA transcription factor
Unigene3706_All	51.60	135.05	1.39	up	3.28E-68	1.09E-65	TGA transcription factor
Unigene11738_All	5.11	21.60	2.08	up	2.73E-09	1.15E-07	Phospholipase A1
Unigene37023_All	27.84	114.64	2.04	up	5.60E-20	5.05E-18	Phospholipase A1
Unigene45678_All	37.39	154.04	2.04	up	1.38E-107	7.9E-105	Lipoxygenase
Unigene11030_All	62.93	152.57	1.28	up	9.16E-44	1.83E-41	Allene oxide cyclase
Unigene29173_All	85.71	199.13	1.22	up	6.06E-72	2.09E-69	12-oxophytodienoic acid reductase
Unigene11800_All	9.14	133.63	3.87	up	2.29E-105	1.25E-102	Jasmonate ZIM domain-containing protein
Unigene19974_All	25.67	112.38	2.13	up	9.06E-53	2.20E-50	Jasmonate ZIM domain-containing protein
Unigene28971_All	32.55	103.54	1.67	up	2.66E-35	4.12E-33	Jasmonate ZIM domain-containing protein
Unigene14746_All	10.23	30.30	1.57	up	8.59E-10	3.78E-08	MYC2 transcription factor
Unigene19948_All	25.16	67.21	1.42	up	2.18E-25	2.48E-23	MYC2 transcription factor
Unigene17336_All	23.29	56.14	1.27	up	4.61E-23	4.76E-21	MYC2 transcription factor
Unigene28993_All	38.28	88.92	1.22	up	2.33E-18	1.89E-16	MYC2 transcription factor
Unigene3689_All	60.21	26.95	−1.16	down	2.97E-26	3.47E-24	MYC2 transcription factor
Unigene10068_All	55.68	122.23	1.13	up	8.46E-10	3.73E-08	1-aminocyclopropane-1-carboxylate synthase
Unigene38824_All	58.78	121.12	1.04	up	1.10E-08	4.32E-07	1-aminocyclopropane-1-carboxylate synthase
Unigene1735_All	99.27	203.73	1.04	up	8.39E-15	5.38E-13	1-aminocyclopropane-1-carboxylate synthase
Unigene23619_All	12.85	57.09	2.15	up	2.21E-26	2.59E-24	DELLA protein
Unigene21755_All	9.62	43.11	2.16	up	9.28E-12	4.81E-10	DELLA protein
Unigene29632_All	99.04	300.15	1.60	up	6.89E-90	3.08E-87	DELLA protein
Unigene41060_All	39.09	110.66	1.50	up	6.35E-12	3.35E-10	DELLA protein
Unigene21602_All	24.78	54.70	1.14	up	1.41E-28	1.81E-26	DELLA protein

The criteria used for assigning significance were: *P*-value < 0.05, FDR ≤ 0.001, and absolute |log_2_Ratio(Y/CK)| ≥ 1. CK: control; Y: aphid infestation treatment.

**Table 3:** **Differentially expressed genes (DEGs) involved in phytohormone metabolism and signaling pathway in the comparison between CK and Z (CK-VS-Z)**

GeneID	CK-RPKM	Z-RPKM	log _2_Ratio (Z/CK)	Up-Down-Regulation (Z/CK)	P-value	FDR	Gene description
Unigene107_All	6.80	19.73	1.54	up	2.95E-12	2.70E-10	NPR1-1 protein
Unigene23699_All	23.61	56.87	1.27	up	2.04E-15	2.36E-13	NIM1-like protein 1
Unigene15228_All	70.35	150.72	1.10	up	9.75E-47	3.75E-44	Phospholipase A1
Unigene37023_All	27.84	65.81	1.24	up	1.20E-06	5.99E-05	Phospholipase A1
Unigene45678_All	37.39	109.57	1.55	up	1.01E-53	4.64E-51	Lipoxygenase
Unigene752_All	1.22	8.31	2.77	up	1.65E-05	0.000669	Lipoxygenase
Unigene26067_All	33.48	95.98	1.52	up	2.99E-72	1.99E-69	Allene oxide synthase
Unigene11030_All	62.93	216.68	1.78	up	1.32E-100	1.49E-97	Allene oxide cyclase
Unigene29173_All	85.71	194.21	1.18	up	7.99E-68	4.81E-65	12-oxophytodienoic acid reductase
Unigene45901_All	14.60	58.94	2.01	up	1.30E-30	3.00E-28	12-oxophytodienoic acid reductase
Unigene11800_All	9.14	169.72	4.21	up	3.46E-143	6.12E-140	Jasmonate ZIM domain-containing protein
Unigene19974_All	25.67	146.79	2.52	up	3.21E-84	2.52E-81	Jasmonate ZIM domain-containing protein
Unigene28971_All	32.55	105.47	1.70	up	4.12E-37	1.16E-34	Jasmonate ZIM domain-containing protein
Unigene21174_All	36.91	120.43	1.71	up	2.79E-50	1.22E-47	Jasmonate ZIM domain-containing protein
Unigene14746_All	10.23	51.02	2.32	up	1.74E-25	3.29E-23	MYC2 transcription factor
Unigene19948_All	25.16	121.61	2.27	up	6.16E-85	4.98E-82	MYC2 transcription factor
Unigene17336_All	23.29	60.28	1.37	up	6.73E-28	1.40E-25	MYC2 transcription factor
Unigene28993_All	38.28	106.40	1.47	up	6.39E-29	1.37E-26	MYC2 transcription factor
Unigene23619_All	12.85	27.10	1.08	up	9.78E-06	0.000418	DELLA protein

The criteria used for assigning significance were: *P*-value < 0.05, FDR ≤ 0.001, and absolute |log_2_Ratio(Z/CK)| ≥ 1. CK: control; Z: mock puncture treatment.

**Table 4:** **Differentially expressed** ***WRKY*** **,** ***MYB, AP2/ERF*** **,** ***GRAS*** **and** ***HSF*** **genes responding to aphid herbivory in the comparison between CK and Y (CK-VS-Y)**

GeneID	CK-RPKM	Y-RPKM	log _2_Ratio(Y/CK)	Up-Down-Regulation(Y/CK)	P-value	FDR	Gene description
Unigene12209_All	20.74	92.22	2.15	up	3.73E-45	7.69E-43	WRKY transcription factor
Unigene41938_All	43.08	183.74	2.09	up	9.59E-37	1.56E-34	WRKY transcription factor
Unigene32329_All	10.38	43.97	2.08	up	2.25E-08	8.60E-07	WRKY transcription factor
Unigene10297_All	85.74	348.25	2.02	up	3.24E-56	8.40E-54	WRKY transcription factor
Unigene37863_All	85.47	340.39	1.99	up	7.04E-94	3.33E-91	WRKY transcription factor
Unigene20571_All	20.38	73.31	1.85	up	2.52E-19	2.19E-17	WRKY transcription factor
Unigene37259_All	17.93	64.06	1.84	up	9.77E-11	4.64E-09	WRKY transcription factor
Unigene37869_All	268.18	945.12	1.82	up	1.82E-155	2.00E-152	WRKY transcription factor
Unigene7360_All	74.03	251.85	1.77	up	6.60E-41	1.20E-38	WRKY transcription factor
Unigene37669_All	337.88	1022.27	1.60	up	9.04E-90	4.01E-87	WRKY transcription factor
Unigene1677_All	38.23	115.12	1.59	up	3.73E-18	2.99E-16	WRKY transcription factor
Unigene17473_All	33.01	69.77	1.08	up	1.31E-07	4.55E-06	WRKY transcription factor
Unigene6575_All	23.05	118.37	2.36	up	3.80E-48	8.14E-46	MYB transcription factor
Unigene27371_All	35.74	136.17	1.93	up	5.40E-40	9.55E-38	MYB transcription factor
Unigene29130_All	35.63	95.08	1.42	up	1.66E-37	2.74E-35	MYB transcription factor
Unigene5110_All	46.71	113.33	1.28	up	7.40E-29	9.65E-27	MYB transcription factor
Unigene20732_All	27.31	61.70	1.18	up	2.12E-12	1.18E-10	MYB transcription factor
Unigene1509_All	21.86	48.30	1.14	up	2.69E-07	9.03E-06	MYB transcription factor
Unigene10992_All	29.16	8.50	−1.78	down	2.82E-16	2.01E-14	MYB transcription factor
Unigene33772_All	33.67	115.91	1.78	up	1.32E-15	9.07E-14	AP2/ERF transcription factor
Unigene29332_All	26.35	85.91	1.71	up	2.06E-18	1.68E-16	AP2/ERF transcription factor
Unigene37496_All	281.36	569.19	1.02	up	3.06E-30	4.18E-28	AP2/ERF transcription factor
Unigene20692_All	107.36	250.56	1.22	up	6.69E-43	1.30E-40	AP2/ERF transcription factor
Unigene28929_All	196.76	405.51	1.04	up	1.12E-67	3.67E-65	AP2/ERF transcription factor
Unigene20430_All	11.14	71.20	2.68	up	4.27E-43	8.31E-41	AP2/ERF transcription factor
Unigene21602_All	24.78	54.70	1.14	up	1.41E-28	1.81E-26	GRAS transcription factor
Unigene23619_All	12.85	57.10	2.15	up	2.21E-26	2.59E-24	GRAS transcription factor
Unigene11471_All	9.76	31.50	1.69	up	5.41E-07	1.74E-05	GRAS transcription factor
Unigene41060_All	39.09	111.00	1.50	up	6.35E-12	3.35E-10	GRAS transcription factor
Unigene24298_All	32.99	76.36	1.21	up	6.17E-23	6.34E-21	Heat shock factor
Unigene3496_All	27.02	104.20	1.95	up	2.08E-49	4.55E-47	Heat shock factor
Unigene24225_All	14.71	5.05	−1.54	down	1.58E-07	5.46E-06	Heat shock factor

The criteria used for assigning significance were: *P*-value < 0.05, FDR ≤ 0.001, and absolute |log_2_Ratio(Y/CK)| ≥ 1. CK: control; Y: aphid infestation treatment.

**Table 5:** **Differentially expressed** ***WRKY*** **,** ***MYB, AP2/ERF*** **,** ***GRAS*** **and** ***HSF*** **genes responding to aphid herbivory in the comparison between CK and Z (CK-VS-Z)**

GeneID	CK-RPKM	Z-RPKM	log _2_Ratio(Z/CK)	Up-Down-Regulation(Z/CK)	P-value	FDR	Gene description
Unigene26514_All	21.32	50.79	1.25	up	4.13E-06	0.000192	WRKY transcription factor
Unigene20571_All	20.38	46.36	1.19	up	2.41E-07	1.31E-05	WRKY transcription factor
Unigene29130_All	35.63	82.07	1.20	up	5.25E-26	1.02E-23	MYB transcription factor
Unigene33100_All	10.52	43.44	2.05	up	6.04E-19	8.69E-17	MYB transcription factor
Unigene33772_All	33.67	88.24	1.39	up	5.09E-09	3.43E-07	AP2/ERF transcription factor
Unigene29332_All	26.35	59.86	1.18	up	2.45E-08	1.52E-06	AP2/ERF transcription factor
Unigene20430_All	11.14	66.16	2.57	up	7.72E-39	2.25E-36	AP2/ERF transcription factor
Unigene23619_All	12.85	27.10	1.08	up	9.78E-06	0.000418	GRAS transcription factor
Unigene3496_All	27.02	62.69	1.21	up	3.72E-16	4.59E-14	Heat shock factor

The criteria used for assigning significance were: *P*-value < 0.05, FDR ≤ 0.001, and absolute |log_2_Ratio(Z/CK)| ≥ 1. CK: control; Z: mock puncture treatment.

### Quantitative real-time PCR (qRT-PCR) validation of differentially expressed genes (DEGs) from RNA-Seq

**Figure 6:**
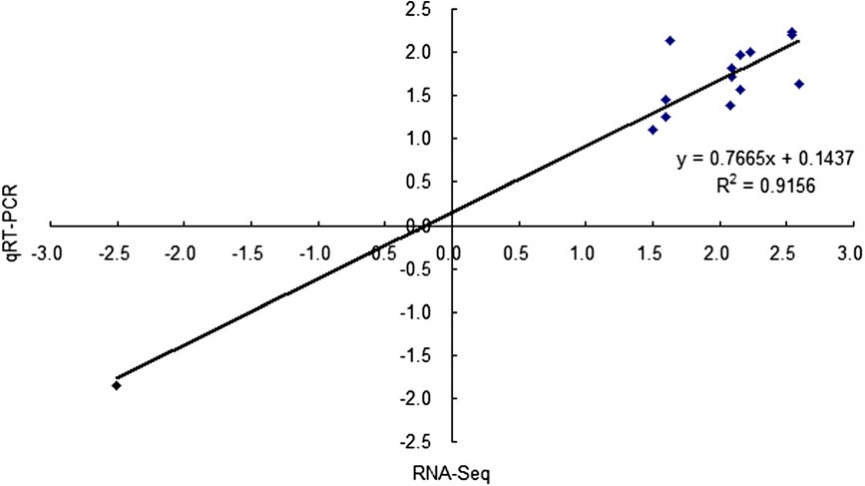
**Quantitative real-time PCR (qRT-PCR) validation of differentially expressed genes (DEGs) from RNA-Seq in leaf tissues of chrysanthemum.** Correlation of fold change analyzed by RNA-Seq platform (*x* axis) with data obtained using qRT-PCR (*y* axis).

To validate the results of Illumina RNA-Seq, several genes from library CK and Y (CK: control; Y: aphid infestation treatment) were chosen randomly for qRT-PCR. For comparison of fold change between RNA-Seq and qRT-PCR, scatterplots were generated using the log_2_ fold change determined by RNA-Seq and qRT-PCR. As shown in Figure 6, the qRT-PCR results revealed that the expression tendency of these genes showed significant similarity (*r*^*2*^ = 0.92) with the Illumina RNA-Seq data, suggesting the reproducibility and accuracy of RNA-Seq results.

## Discussion

RNA sequencing technology allows us to have a comprehensive view on the gene expression changes induced by aphids. And there are numerous genes whose expressions are changed after aphid feeding. Here, we mainly focus on the discussion on genes related to phytohormone signaling pathways and aphid feeding-associated transcription factors (TFs), photosynthesis, reactive oxygen species (ROS), cell wall biosynthesis and nucleotide-binding-site leucine-rich-repeat (NBS-LRR) genes.

### Plant hormone signaling pathway involved in plant-aphid interaction

Salicylic acid (SA), jasmonic acid (JA) and ethylene (ET) are three major phytohormones reported in the regulation of signaling networks involved in aphid-induced defense responses. SA is important for localized plant tissue hypersensitive responses (HR), and could activate systemic acquired resistance (SAR), which is a broad-spectrum resistance of plants and it is necessary to transduce SA signal to stimulate the transcription of defense response genes, such as *pathogenesis-related* (*PR*) genes [18, 22, 23]. *Non-expressor of pathogenesis-related genes1* (*NPR1*), also called *non-inducible immunity1* (*NIM1*), is a key factor of SAR, and activates the expression of *PR* genes upon binding to TGAs, transcription factors which bind to SA-responsive elements (TGACG) in the promoters of *PR* genes [24]. Besides its regulatory role in *PR* gene expression, *NPR1* also participates in the inhibition of JA signaling by SA [25]. In the present study, three *NPR1* genes (Unigene107_All, Unigene23699_All and Unigene16290_All) and two *TGA* genes (Unigene2058_All and Unigene3706_All) were up-regulated by aphid infestation in the CK and Y comparison (CK-VS-Y) (Table 2). Two *NPR1* genes (Unigene107_All and Unigene23699_All) was induced by mock puncture treatment in the CK and Z comparison (CK-VS-Z) (Table 3), implying that Unigene16290_All might respond specifically to aphid feeding. In *Arabidopsis thaliana*, *npr1* and *nim1* mutant plants are deficient in SA-induced disease resistance [24]. Further study suggests that the cytosolic function of NPR1 plays a role in SA-JA antagonism, and the nuclear function of NPR1 plays a role in the induction of SA-responsive genes [25, 26].

The JA signaling transduction, containing wound hormone jasmonoyl-isoleucine (JA-Ile), is another well studied regulator of plant resistance to aphids [27, 28]. Genes involved in JA synthesis [29], such as *phospholipase*, *lipoxygenase* (*LOX*), *allene oxide synthase* (*AOS*), *allene oxide cyclase* (*AOC*) and *12-oxophytodienoic acid reductase* (*OPR*) were all stimulated after aphid infestation and mock puncture treatment in the CK-VS-Y and CK-VS-Z (Tables 2 and 3). Several previous studies have indicated the roles of JA in aphid infestation responses, for example, *LOX* genes were strongly up-regulated by *Myzus persicae* feeding on *A. thaliana* leaves [9], *M. nicotianae* feeding on *Nicotiana attenuata* leaves [30], and *M. euphorbiae* on tomato leaf tissues [31]. Infestation of potato (*Solanum tuberosum* L.) by *M. persicae* induced transcripts encoding *PR-1*, which increased gradually during the time-course of aphid feeding, and the expression of *JAZ1* was kept at a stable level [32]. In present study, three *JAZ* (Unigene11800_All, Unigene19974_All and Unigene28971_All) and five *MYC2* genes (Unigene14746_All, Unigene19948_All, Unigene17336_All, Unigene28993_All and Unigene3689_All) were significantly differentially expressed in CK-VS-Y (Table 2). There were four differentially expressed *JAZ* (Unigene11800_All, Unigene19974_All, Unigene28971_All and Unigene21174_All) and four *MYC2* genes (Unigene14746_All, Unigene19948_All, Unigene17336_All and Unigene28993_All) in CK-VS-Z (Table 3), indicating that Unigene3689_All might be related with the JA signaling pathway and play a major role in wound-induced response by aphid infestation. Jasmonate ZIM-domain proteins (JAZ) identified as key players of JA signaling cascade repress expression of JA-responsive genes by binding to transcriptional factors, such as MYC2 [33]. Plants increase the synthesis of JA which is then transformed to JA-Ile by jasmonic acid resistant 1 (JAR1) enzyme under stress [34]. The JA-Ile conjugate promotes interaction between JAZ and COI1 proteins in Skp/Cullin/F-box complex (SCF^COI1^), resulting the degradation of JAZ through SCF^COI1^-dependent 26S proteasome pathway and the removal of inhibition to MYC2, thereby starting the transcription of JA-responsive genes, such as *vegetative storage protein* (*VSP*) gene [35–37]. How the JAZ and MYC2 regulate the response of chrysanthemum to aphid infestation is to be studied further.

Though relatively few studies have been reported on the participation of ET in plant-aphid interactions, some studies have suggested that aphid infestation markedly increased the production of ET in leaves of plants, including barley [16], celery [38], Arabidopsis [19] and wheat [39]. Unigene10068_All, Unigene38824_All and Unigene1735_All encoding 1-aminocyclopropane-1-carboxylic acid (ACC) synthases, the key enzymes in ET biosynthesis, were up-expressed in the CK-VS-Y (Table 2). ETHYLENE INSENSITIVE2 (EIN2), a membrane protein, plays an essential role in ET signaling pathway and is indispensable for defense responses. For instance, the *EIN2* gene is demanded for the induced resistance to *M. persicae* in Arabidopsis treated by HrpN_Ea_ [40]. Besides, ET signaling pathway through EIN2 results in transcription of the plant defensin gene *PDF1.2*, a molecular marker of ET signal transduction and needs *EIN2* for transcription [40]. ET often works synergistically with JA [8]. Recent study shows that the ET-stabilized transcriptional factors (EIN3/EIL1) mediate several ET transcriptional responses that are regulated by crosstalk with JA, which enhances the activity of EIN3/EIL1 by removal of JAZ proteins repressing EIN3/EIL1 [41]. However, there is no *EIN2*, *PDF1.2* or *EIN3/EIL1* gene significantly differentially expressed in both CK-VS-Y and CK-VS-Z comparison, which may be related with the insensitivity of chrysanthemum to ethylene [42, 43], therefore, we suggested that the insensitivity of chrysanthemum to ET might partially compromise ET cascade or ET-mediated aphid infestation response in chrysanthemum in a different way from that in other plants.

Besides the SA-JA-ET backbone, other plant hormones, such as abscisic acid (ABA), auxin and gibberellin (GB), have gotten less attention as potential factors that mediate aphid resistance. However, these hormones also play a significant role in herbivore-induced defense responses, feeding into the SA-JA-ET network. ABA synthesis and cascades affect herbivore-activated JA metabolism and signaling in Arabidopsis [44], maize [45] and tomato [46]. Synergy between ABA and JA could stimulate MYC-dependent gene expression [47], and MYC2 functions as an integration point between the ABA and JA pathways [48, 49]. Auxin and JA co-regulate JAZ1 and MYC2 [50, 51]. Interestingly, gibberellic acid (GA) affect the JA signaling pathway through competitively binding to JAZ proteins instead of DELLAs, negative regulators of GB signaling, thereby promoting MYC2-induced gene expression [52]. GA perception results in degradation of DELLAs, leading to the inhibition of MYC2 and attenuated JA responses. The expression of five *DELLA* genes (Unigene23619_All, Unigene21755_All, Unigene29632_All, Unigene41060_All and Unigene21602_All) was modulated by aphid infestation in CK-VS-Y (Table 2) and one gene (Unigene23619_All) by mock puncture experiment in CK and Z (Table 3), indicating the complex connections between different plant hormone signalings induced by aphid in chrysanthemum leaf.

### Transcription factors (TFs) responding to aphid infestation

TFs are important regulators of plants’ defense response. Several members of TF families have been reported to be involved in plant-herbivore interaction. Overexpression of *OsWRKY89* increased the resistance of rice to white-backed planthopper, *Sogatella furcifera*, a sap-sucking insect [53]. In *Nicotiana attenuata*, silencing *WRKY3* and/or *WRKY6* makes plants more susceptible to insect herbivory, and this susceptibility is connected with the impairment of JA accumulation and defenses mediated by JA signaling [54], suggesting the crosstalk between TFs and phytohormone signaling. Silencing and overexpression of *OsERF3* indicate that the gene is a central early herbivore-responsive one that affects a set of defense-associated signaling pathways, such as MAPK cascades as well as SA, JA and ET signaling, and it acts as a vital switch modulating defense responses against chewing and piercing/sucking insects in rice [55]. The aphid-susceptible *atmyb44* mutant showed a much greater susceptibility to aphid feeding and most compromised in induced resistance in Arabidopsis. A further stud shows that *atmyb44* incurred an abolishment of the induction of *EIN2*, indicating a close link between *AtMYB44* and *EIN2* [40]. Besides their direct functions in plant-aphid interaction, TFs may also regulate the growth and development of plants to mediate defense responses indirectly, including photosynthesis, cell wall formation, carbon metabolism and so on. In present study, we also identified several differentially expressed TFs that were reported previously, including *WRKY*, *MYB* and *AP2/ERF*, and some new TFs responding to aphid herbivory in chrysanthemum, such as *GRAS* and *HSF* genes (Tables 4 and 5). In the CK-VS-Y, twelve *WRKY*, seven *MYB*, six *AP2/ERF*, four *GRAS* and three *HSF* genes were recognized, whereas only two *WRKY* genes, two *MYB* genes, three *AP2/ERF* genes, one *GRAS* genes and one *HSF* genes were identified in the CK-VS-Z, implying that aphid feeding has bigger influences on gene expression and is more complicated than mock puncture treatment, and the new discovered aphid-responsive TFs, *GRAS* (Unigene21602_All, Unigene11471_All and Unigene41060_All) and *HSF* genes (Unigene24298_All and Unigene24225_All), might express specifically to aphid infestation. Still, the potential roles of these TFs need further investigation.

### Reactive oxygen species (ROS) and antioxidant genes

Besides being toxic byproducts of metabolism, ROS, for example hydrogen peroxide (H_2_O_2_), are also involved in the complex signaling network of plants [56, 57]. There are at least three possible roles for ROS in plant-aphid interaction: direct adverse influences on aphid midgut tissues [8], triggering programmed cell death (PCD) [58] leading to apoptosis to against biotrophic aphids and stimulating defense signaling pathways towards aphid attack [56]. Aphid feeding alters plant redox state and induces the production of ROS [8], and others could also elicit the accumulation of ROS content, such as SA and JA, indicating possible interactions between ROS signaling and phytohormone transduction. Research of Russian wheat aphid [59], *Diuraphis noxia* (Mordvilko) infestation on wheat (*Triticum aestivum* L.) resulted in induction of H_2_O_2_ content and activity of NADPH oxidase from which ROS are largely derived [60], and strongly indicated a probable signaling role for H_2_O_2_. Here, three NADPH oxidase genes, Unigene45792_All, Unigene300_All and Unigene3581_All, were recognized in CK and Y comparison alone (Additional file 8: Table S7). Furthermore, enzymes, such as peroxidase (POD), ascorbate peroxidase (APX) and polyphenol oxidase (PPO), involved in ROS scavenging were also up-regulated during aphid infestation (Additional file 8: Table S7 and Additional file 9: Table S8), suggesting the maintenance of redox homeostasis is important for responses to aphid, which are consistent with our previous observation of the enhanced enzyme activities by aphid infestation. Except linked with detoxification of ROS, enzymes, such as peroxidases, are yet prerequisites for plant cell wall building [61], further demonstrating the complex regulatory network inside plants.

### Photosynthesis-associated genes involved in response to aphid feeding

Aphids, phloem-feeding herbivores, drain plant nutrients of which the main components are saccharides resulting from photosynthesis. Saccharides drained from the sieve element are easy to be contaminated by bacteria on the surface of leaves, thereby affecting photosynthesis. In our study, only two photosynthesis-related genes (Unigene24131_All and Unigene9460_All) were detected in the CK and Y alone (Additional file 14: Table S13), both of them belonging to the components of photosystem were induced by aphids, which may suggest the strengthening of photosynthesis, compensating for the loss of nutrients and maintaining the normal growth processes. *D. noxia* feeding on leaves of wheat [39], *M. persicae* feeding on celery foliage [38] and *M. nicotianae* feeding on *N. attenuata* leaves tissues [30] promote the expression of photosynthesis genes, while some of them are decreased by *M. nicotianae* [30] or *Schizaphis graminum* [62], possibly reflecting the redistribution of metabolites from normal growth functions to defensive roles after aphids feeding in plants.

### Nucleotide-binding site-leucine-rich repeat (NBS-LRR) genes

Two cloned aphid resistance (*R*) genes, *Mi-1.2*, conferring resistance to the potato aphid, *Macrosiphum euphorbiae* (Thomas) [3, 4], and *Vat*, mediating resistance to the cotton aphid, *Aphis gossypii* Glover [5, 63], belong to NBS-LRR family. Similarly, other plant-aphid interactions have revealed a tight relationship between NBS-LRR genes and resistance loci. Plants of wheat having *D. noxia* resistance gene contain leucine zipper (LZ)-NBS-LRR sequences [64–66]. Swanepoel and co-workers [67] also discovered tight connection between LZ-NBS-LRR sequence and *D. noxia* resistance gene. On the chromosome of *Medicago truncatula*, a locus which controls the resistance to the blue alfalfa aphid, *Acyrthosiphon kondoi*, is flanked by coiled-coil (CC)-NBS-LRR sequence [7]. Similarly, we found two differentially expressed genes (Unigene3633_All and Unigene14351_All) containing NBS-LRR region in CK-VS-Y (Additional file 15: Table S14). Further cloning and functional identification regarding the two genes would be necessary.

### Genes involved in cell wall biosynthesis

In Arabidopsis, several *COBRA* and *COBRA-like* genes have been identified to be important for secondary cell wall development [68]. Loss of function mutation of these genes results in brittle stalks and decreased cellulose content [69], indicating that these genes are essential for normal cellulose deposition in secondary cell wall. Mutations in *brittle culm1* (*bc1*) which encodes a COBRA-like protein suggest that it controls the mechanical strength of monocots and is an important player in the biosynthesis of cell walls of mechanical tissues [70]. There are three *COBRA-like* genes (Unigene11326_All, Unigene2724_All and Unigene22759_All) identified in CK-VS-Y, and two (Unigene11326_All and Unigene2724_All) out of three in CK-VS-Z (Additional file 10: Table S9 and Additional file 11: Table S10). Hemicelluloses and pectins, which are both synthesized in the Golgi, and cellulose and callose, both synthesized at plasma membrane, are the major polysaccharides of the plant cell wall. The identification of cellulose synthase A (CesA), which is the catalytic subunit of the cellulose synthase complex [71, 72], greatly enriches our understanding of the biosynthesis of cell wall polysaccharides. And some *cellulose synthase-like* (*Csl*) genes have also been reported to be responsible for the biosynthesis of glycan backbones in the Golgi [73]. In this study, two (Unigene25922_All and Unigene6200_All) and three *Csl* genes (Unigene3108_All, Unigene25922_All and Unigene6200_All) were detected in the CK-VS-Y and CK-VS-Z, respectively (Additional file 10: Table S9 and Additional file 11: Table S10). The up-regulation of *COBRA-like* and *Csl* genes suggests that the mechanical strength of the plant are somewhat strengthened, which might therefore hinder the puncturing of the aphid stylet during aphid feeding. Therefore, the detailed mechanisms of these genes during plant-aphid interactions could be another interesting topic, and relevant transgenic work would be more practical.

### Secondary metabolites

Secondary metabolites, such as flavonoids, terpenes, phenolics and alkaloids, having antixenotic or antibiotic properties, could function in plant defense against herbivores [74]. In *Vigna* [75], there is a positive relationship between resistance or susceptibility properties against aphids and flavonoid glycoside content. The content of flavonoid in susceptible lines was lower than in resistant ones. *In vitro* bioassays proved that quercetin and isorhamnetin, members of endogenous flavonoids, have a significant inhibitory on the reproduction rate of aphids. In contrast, overexpression of *AtMYB75*, resulting in increasing flavonol levels, did enhance the resistance to caterpillars, but with no effects on the performance of *B. brassicae* [76]. Flavonoids, including flavones and isoflavones [77], are derived from the phenylpropanoid pathway, which is catalyzed by a number of enzymes, for example, PAL (phenylalanine ammonia-lyase), which is well studied for plant responses to biotic and abiotic stress. In this study, we got several DEGs related with flavonoids synthesis, such as PAL, in both CK-VS-Y and CK-VS-Z (Additional file 12: Table S11 and Additional file 13: Table S12). Attacked by herbivores, some plants would emit volatile compounds, which are mainly mono- and sesquiterpenes, used by parasitic wasps to find their hosts, the lepidopteran larvae. Terpene synthases catalyze the committed step in the biosynthesis of varieties of mono- and sesquiterpene products from prenyl diphosphate precursors. The expression of *terpene synthase 1* (*tps1*) in the maize cv B73 was stimulated by herbivory and mechanical damage. Further analysis shows that the transcription of *tps1* or its homolog varies between different cultivars of maize [78]. Our previous study found that the increased content of monoterpenoids and sesquiterpenoids in the leaves of the hybrid between chrysanthemum and *Artemisia vulgaris* enhanced plant resistance to aphid [79]. Interestingly, two terpene synthase encoding genes (Unigene3919_All and Unigene26695_All) were detected in CK-VS-Y and CK-VS-Z, respectively (Additional file 12: Table S11 and Additional file 13: Table S12). These discussed above illustrate the involvement of secondary metabolites during aphid herbivory in chrysanthemum leaf, indicating their potential roles in the defense responses against aphids.

### Aphid feeding and mock puncture treatment

Here, in our research, we conducted a mock puncture treatment trying to partially simulate the mechanical stress resulting from aphid penetration. Despite there are some differences between aphid stylet and puncture. For instance, aphid stylets were often wrapped by saliva which contains a complex mixture of enzymes and can induce defense responses [80]. Also, the mechanical degree of puncture treatment should be different from aphid stylets. Results that were discussed above show that it does have some similarities between aphid feeding and puncture treatment, such as genes involved in phytohormone metabolism and signaling pathway, ROS scavenging and cell wall biosynthesis, and some genes specifically expressed in response to aphid treatment, for example, NBS-LRR genes. And as shown in Figure 4B, 648 DEGs were specifically expressed in CK-VS-Y; 328 DEGs were co-expressed in CK-VS-Y and CK-VS-Z, suggesting that genes co-expressed in response to aphid feeding and puncture treatment might be involved in wound-induced response by aphid, otherwise genes may specifically respond to aphid sucking. These will allow us to figure out the potential impacts of aphid stylets and refine the processes of defense responses.

## Conclusions

Taken together, these examples indicate that aphid feeding does have a global effect on gene expression in chrysanthemum leaf, including genes involved in phytohormone signaling, cell wall biosynthesis, photosynthesis, reactive oxygen species (ROS) pathway and transcription factors (TF), and so on. Usually, there are cross-communications between different defense pathways those genes belonging to, which provide an ability that allows plants to integrate environmental, developmental and defense-related signals, fine-tuning its defense responses.

## Methods

### Plants growth

*Chrysanthemum morifolium* ‘nannongxunzhang’ (aphid resistant) was obtained from the Chrysanthemum Germplasm Resource Preserving Centre, Nanjing Agricultural University, China. Seedlings were grown in 12 cm pots with a 1:2 mixture of vermiculite and garden soil without fertilizer. Plants were grown under a 16 h photoperiod (160 *μ* mol m^−2^ s^−1^ photon flux density), a relative humidity of 80%, and a day/night temperature of 25/18°C in a greenhouse. Uniformity plants grown to the 6–8 leaf stage were selected for further experiment.

### Aphid infestation and mock puncture treatment

Aphids (*Macrosiphoniella sanbourni* Gillette) were collected from field-grown chrysanthemum plants, two instars nymphs were fostered and chosen to inoculate plants. For aphid infestation treatment (Y), the third fully expanded leaves from stem tip were infested with twenty second instar aphids transferred by a soft brush. The infested leaves were caged with transparent ventilated plastic cages (2 cm height × 5 cm diameter) sealed at the base of the petiole, equal to the leaves of control (CK) and mock puncture treatment (Z). For the mock puncture treatment (Z), designed to partially simulate the mechanical stress resulting from aphid penetration, the third fully expanded leaf of each plant was punctured 5 times at 0 h, 10 times at 24 h, and 15 times at 48 h with a needle (approximately 0.30 mm diameter) [9]. Leaves of three seedlings for each treatment were harvested at 0 h, 3 h, 6 h, 12 h, 24 h, 48 h. Before harvest, aphids were removed by spraying with 1% (v/v) SDS solution, which caused aphids to remove their mouthparts from plant tissues and then removed the aphids from the leaves by flushing the plants with deionized water. Harvested materials were immediately frozen in liquid nitrogen and stored at −80°C for the following experiments. The samples collected at defined time points of each treatment were pooled for RNA-Seq.

### RNA extraction, cDNA library construction and Illumina sequencing

Total RNA from leaf tissue of three separate libraries (CK, Z, Y) was extracted using RNAiso reagent (TaKaRa, Japan), following the manufacturer’s instructions. The integrity and quality of the total RNA was evaluated using a 2100 Bioanalyzer RNA Nano chip device (Agilent, Santa Clara, CA, USA) and agarose gel electrophoresis, and the concentration was measured with a ND −1000 spectrophotometer (NanoDrop, Wilmington, DE).

The mRNA of each library was enriched using poly(T) oligonucleotide-attached magnetic beads. Following purification, the mRNA was fragmented to a size of ~200 bp, and the RNA fragments were copied into first-strand cDNA using random hexamer-primed reverse transcription. Second-strand cDNA synthesis was generated using RNaseH and DNA polymerase I, and the cDNA fragments were processed for end repair, an addition of a single “A” base, and ligation of the adapters following Illumina’s protocols and sequenced on Illumina HiSeq^TM^ 2000 platform.

### Processing of sequence data

The raw reads from Illumina sequencing were initially processed to remove adaptor sequences and low-quality reads. The remaining reads called clean reads were then mapped to the set of chrysanthemum unigene sequences using SOAPaligner/SOAP2. No more than two mismatches were allowed for alignment. RPKM (reads per kb per million reads) was used to describe the expression levels of genes. Differential expression of the three libraries was based on the log_2_ ratio of the RPKM values. FDR (false discovery rate) providing a criterion to determine the *P*-value threshold in multiple tests and analyses was also applied to identify differential expressed genes. A stringent cutoff, the *P*-value < 0.05, the FDR ≤ 0.001 and |log_2_Ratio| ≥ 1.0, was used for determining differential expressed genes. Gene ontology (GO) was used to describe the function of these genes, and a hypergeometric test was used to map them to GO terms based on the BGI WEGO (Web Gene Ontology Annotation Plot, http://wego.genomics.org.cn/cgi-bin/wego/index.pl). All sequencing data have been deposited at the sequence read archive (SRA) of NCBI.

### Quantitative real-time PCR (qRT-PCR) validation

qRT-PCR was carried out using a Eppendorf AG 22331 Hamburg thermocycler. The samples collected at different time points were pooled. Three independent biological replicates of each sample and three technical replicates of each biological replicate were used for qRT-PCR analysis. For each sample, 1 ug of total RNA removed DNA by RNase-free DNase I treatment was converted into cDNA using a Super RT kit (BioTeke, Beijing, China). And qRT-PCR was performed in a 20 ul volume containing 10 ul SYBR Green PCR master mix (TaKaRa, Japan), 0.2 uM of each primer (Table 6) and 10 ng cDNA, and the amplification programme including an initial denaturation at 95°C for 60 s, followed by 40 cycles of 95°C for 15 s, 55°C for 15 s and 72°C for 20 s). At the end of the cycling process, a melting-curve analysis from 55 to 95°C with a heating rate of 0.5°C s^−1^ was performed to determine specificity of amplified products. The chrysanthemum *EF1α* gene was used as a reference. Relative expression levels were calculated using the 2-^ΔΔCT^ method.

**Table 6:** **Primers of quantitative real-time PCR (qRT-PCR) validation of differentially expressed genes (DEGs)**

Gene ID	Forward primer	Reverse primer	Annotation
Unigene12209_All	GTGGCTGAGATTGGTGGTTT	GCCTTTACAAGCGTTTCAGC	WRKY family transcription factor
Unigene14378_All	TTAAGTCGGTTTTCGGCTTG	GCATCCTCTTCGATCCTTTG	WRKY family transcription factor
Unigene14705_All	GACCGTCAAGAACAGGGGTA	ATAGAAGGTCCCGCAAACCT	Protein kinase
Unigene23047_All	GCCACAAAATCCGTCAACTT	GCCTAACGATCCCTTGTGAA	SAUR family gene
Unigene22169_All	GTCAAATGCTGCAAGGGATT	ATCAACACTTGCCCGAAGAC	Disease resistance protein
Unigene22508_All	CGCGTTTCTTTCATTCCATT	CGGTCGAACCCAGATTTAAG	Kinase
Unigene32329_All	TCGTACCGCTGGGAATTTAG	TGGGCTCGACTCGACTACTT	WRKY family transcription factor
Unigene23619_All	ATGGGTGTTACAGGGATGGA	ACACAGGAGAGCTCCAGGAA	GRAS family transcription factor
Unigene29632_All	CCTCCTAAGCTTCGCATCAC	GCTGTTAACCGCTGACCAAT	gibberellin-responsive protein
Unigene41060_All	GTAATCTGGAGCATGGGTGG	CTTAATGGTGTGCCCGTTTC	GRAS family transcription factor
Unigene36228_All	GGTTGTTTGGTTCTCGGAAA	TACCAACAGTAACACCGCCA	Protein kinase
Unigene41938_All	GAGGATTTTCGCTGCCTTTA	TCAACCACAAGAATGGAGCA	WRKY family transcription factor
Unigene49088_All	ACACTTTGTTTTCGGTTGGG	GCAAGACCAACCATGAGGAT	Protein kinase
Unigene55750_All	ACCAGGATAAGGGAAGACGG	TCCATCCCAAATTTCCAAAA	protein with unknown function

### Availability of supporting data

The data sets supporting the results of this article are available in the NCBI Sequence Read Archive (SRA) database under accession number SRP042216, http://www.ncbi.nlm.nih.gov/sra/?term=SRP042216.

## Electronic supplementary material

Additional file 1: Figure S1: Component of the raw reads in the three RNA libraries. “Clean reads” are those remaining after removal of adaptor sequences and low-quality reads. The numbers in parentheses indicate the percentage of each type of read present. CK: control; Y: aphid infestation treatment; Z: mock puncture treatment. (TIFF 74 KB)

Additional file 2: Table S1: Differentially expressed genes (DEGs) in the comparison between libraries CK and Y. CK: control; Y: aphid infestation treatment. The criteria used for assigning significance were: *P*-value < 0.05, FDR ≤ 0.001, and estimated absolute |log_2_Ratio(Y/CK)| ≥ 1. Genes listed in descending order of absolute |log_2_Ratio(Y/CK)|. GeneIDs got from the Chrysanthemum Reference Sequence Database. Annotation of unigene sequences performed using BlastX (E < 10). The “GeneLength” column gives the length of exon sequence. CK- and Y-expression: frequency of unigene transcripts in libraries CK and Y, respectively. CK- and Y-RPKM: reads per kb per million reads for each unigene in libraries CK and Y, respectively. Log_2_Ratio(Y/CK): the ratio between the RPKM in Y and the RPKM in CK. KEGG: annotation according to the KEGG database by BLAST. Blast nr: identification of homologues in GenBank. GO Component, GO Function and Go Process: ontology information of Cellular Components, Molecular Function and Biological Processes of Gene-corresponding GO terms. “-”: no hit. (XLS 459 KB)

Additional file 3: Table S2: Differentially expressed genes (DEGs) in the comparison between libraries CK and Z. CK: control; Z: mock puncture treatment. The criteria used for assigning significance were: *P*-value < 0.05, FDR ≤ 0.001, and estimated absolute |log_2_Ratio(Z/CK)| ≥ 1. Genes listed in descending order of absolute |log_2_Ratio(Z/CK)|. GeneIDs got from the Chrysanthemum Reference Sequence Database. Annotation of unigene sequences performed using BlastX (E < 10). The “GeneLength” column gives the length of exon sequence. CK- and Z-expression: frequency of unigene transcripts in libraries CK and Z, respectively. CK- and Z-RPKM: reads per kb per million reads for each unigene in libraries CK and Z, respectively. Log_2_Ratio(Z/CK): the ratio between the RPKM in Z and the RPKM in CK. KEGG: annotation according to the KEGG database by BLAST. Blast nr: identification of homologues in GenBank. GO Component, GO Function and Go Process: ontology information of Cellular Components, Molecular Function and Biological Processes of Gene-corresponding GO terms. “-”: no hit. (XLS 214 KB)

Additional file 4: Table S3: Differentially expressed genes (DEGs) in the comparison between libraries Z and Y. Z: mock puncture treatment; Y: aphid infestation treatment. The criteria used for assigning significance were: *P*-value < 0.05, FDR ≤ 0.001, and estimated absolute |log_2_Ratio(Y/Z)| ≥ 1. Genes listed in descending order of absolute |log_2_Ratio(Y/Z)|. GeneIDs got from the Chrysanthemum Reference Sequence Database. Annotation of unigene sequences performed using BlastX (E < 10). The “GeneLength” column gives the length of exon sequence. Z- and Y-expression: frequency of unigene transcripts in libraries Z and Y, respectively. Z- and Y-RPKM: reads per kb per million reads for each unigene in libraries Z and Y, respectively. Log_2_Ratio(Y/Z): the ratio between the RPKM in Y and the RPKM in Z. KEGG: annotation according to the KEGG database by BLAST. Blast nr: identification of homologues in GenBank. GO Component, GO Function and Go Process: ontology information of Cellular Components, Molecular Function and Biological Processes of Gene-corresponding GO terms. “-”: no hit. (XLS 146 KB)

Additional file 5: Table S4: GO classification of differentially expressed genes (DEGs) in the comparison between library CK and Y. CK: control; Y: aphid infestation treatment. (XLS 56 KB)

Additional file 6: Table S5: GO classification of differentially expressed genes (DEGs) in the comparison between library CK and Z. CK: control; Z: mock puncture treatment. (XLS 34 KB)

Additional file 7: Table S6: GO classification of differentially expressed genes (DEGs) in the comparison between library Z and Y. Z: mock puncture treatment; Y: aphid infestation treatment. (XLS 28 KB)

Additional file 8: Table S7: Differentially expressed NADPH oxidase genes and enzymes involved in reactive oxygen species (ROS) scavenging responding to aphid herbivory in the comparison between CK and Y (CK-VS-Y). The criteria used for assigning significance were: *P*-value < 0.05, FDR ≤ 0.001, and |log_2_Ratio(Y/CK)| ≥ 1. RPKM: reads per kb per million reads. CK: control; Y: aphid infestation treatment. (DOC 44 KB)

Additional file 9: Table S8: Enzymes involved in reactive oxygen species (ROS) scavenging responding to aphid herbivory in the comparison between CK and Z (CK-VS-Z). The criteria used for assigning significance were: *P*-value < 0.05, FDR ≤ 0.001, and |log_2_Ratio(Z/CK)| ≥ 1. RPKM: reads per kb per million reads. CK: control; Z: mock puncture treatment. (DOC 37 KB)

Additional file 10: Table S9: Differentially expressed genes (DEGs) involved in cell wall biosynthesis responding to aphid herbivory in the comparison between CK and Y (CK-VS-Y). The criteria used for assigning significance were: *P*-value < 0.05, FDR ≤ 0.001, and |log_2_Ratio(Y/CK)| ≥ 1. RPKM: reads per kb per million reads. CK: control; Y: aphid infestation treatment. (DOC 34 KB)

Additional file 11: Table S10: Differentially expressed genes (DEGs) involved in cell wall biosynthesis responding to aphid herbivory in the comparison between CK and Z (CK-VS-Z). The criteria used for assigning significance were: *P*-value < 0.05, FDR ≤ 0.001, and |log_2_Ratio(Z/CK)| ≥ 1. RPKM: reads per kb per million reads. CK: control; Z: mock puncture treatment. (DOC 34 KB)

Additional file 12: Table S11: Differentially expressed genes (DEGs) involved in secondary metabolites responding to aphid herbivory in the comparison between CK and Y (CK-VS-Y). The criteria used for assigning significance were: *P*-value < 0.05, FDR ≤ 0.001, and |log_2_Ratio(Y/CK)| ≥ 1. RPKM: reads per kb per million reads. CK: control; Y: aphid infestation treatment. (DOC 40 KB)

Additional file 13: Table S12: Differentially expressed genes (DEGs) involved in secondary metabolites responding to aphid herbivory in the comparison between CK and Z (CK-VS-Z). The criteria used for assigning significance were: *P*-value < 0.05, FDR ≤ 0.001, and |log_2_Ratio(Z/CK)| ≥ 1. RPKM: reads per kb per million reads. CK: control; Z: mock puncture treatment. (DOC 36 KB)

Additional file 14: Table S13.: Differentially expressed photosynthesis-related genes responding to aphid herbivory in the comparison between CK and Y (CK-VS-Y). The criteria used for assigning significance were: *P*-value < 0.05, FDR ≤ 0.001, and |log_2_Ratio(Y/CK)| ≥ 1. RPKM: reads per kb per million reads. CK: control; Y: aphid infestation treatment. (DOC 29 KB)

Additional file 15: Table S14: Differentially expressed nucleotide-binding site-leucine-rich repeat (NBS-LRR) genes responding to aphid herbivory in the comparison between CK and Y (CK-VS-Y). The criteria used for assigning significance were: *P*-value < 0.05, FDR ≤ 0.001, and |log_2_Ratio(Y/CK)| ≥ 1. RPKM: reads per kb per million reads. CK: control; Y: aphid infestation treatment. (DOC 28 KB)
