# Supplementary figures and images for: Gene expression profiles responses to aphid feeding in chrysanthemum (Chrysanthemum morifolium)
Source: BMC Genomics. 2014 Dec 2;15(1):1050. doi: 10.1186/1471-2164-15-1050 (PMC4265409; doi:10.1186/1471-2164-15-1050)

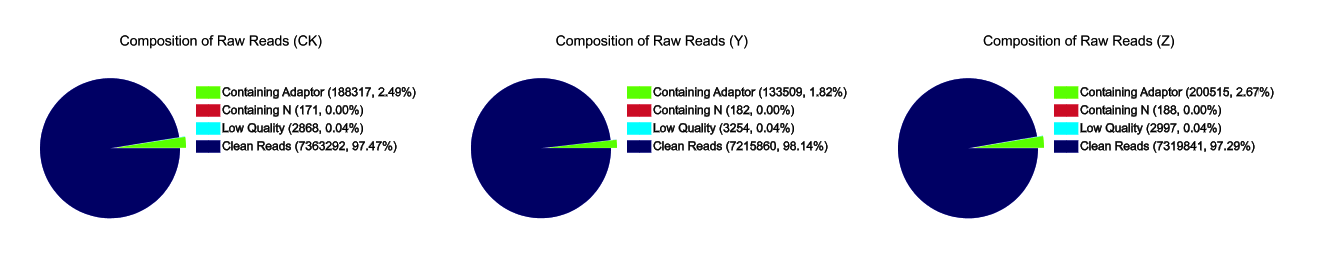

Supplement: Supplementary file 1 — Additional file 1: Figure S1: Component of the raw reads in the three RNA libraries. “Clean reads” are those remaining after removal of adaptor sequences and low-quality reads. The numbers in parentheses indicate the percentage of each type of read present. CK: control; Y: aphid infestation treatment; Z: mock puncture treatment. (TIFF 74 KB) [file 12864_2014_6725_MOESM1_ESM.tiff]
