# Supplementary material for: Gene expression profiles responses to aphid feeding in chrysanthemum (Chrysanthemum morifolium)
Source: BMC Genomics. 2014 Dec 2;15(1):1050. doi: 10.1186/1471-2164-15-1050 (PMC4265409; doi:10.1186/1471-2164-15-1050)
Supplement: Supplementary file 8 — Additional file 8: Table S7: Differentially expressed NADPH oxidase genes and enzymes involved in reactive oxygen species (ROS) scavenging responding to aphid herbivory in the comparison between CK and Y (CK-VS-Y). The criteria used for assigning significance were: P-value < 0.05, FDR ≤ 0.001, and |log2Ratio(Y/CK)| ≥ 1. RPKM: reads per kb per million reads. CK: control; Y: aphid infestation treatment. (DOC 44 KB) [file 12864_2014_6725_MOESM8_ESM.doc]

Additional file 8: Table S7. Differentially expressed NADPH oxidase genes and enzymes involved in reactive oxygen species (ROS) scavenging responding to aphid herbivory in the comparison between CK and Y (CK-VS-Y). The criteria used for assigning significance were: *P*-value < 0.05, FDR ≤ 0.001, and |log_2_Ratio(Y/CK)| ≥ 1. RPKM: reads per kb per million reads. CK: control; Y: aphid infestation treatment.

| GeneID | CK-RPKM | Y-RPKM | log_2_Ratio(Y/CK) | Up-Down-  Regulation(Y/CK) | P-value | FDR | Gene description |
| --- | --- | --- | --- | --- | --- | --- | --- |
| Unigene45792_All | 7.22 | 21.98 | 1.61 | up | 2.99E-07 | 9.98E-06 | NADPH oxidase |
| Unigene300_All | 16.12 | 39.60 | 1.30 | up | 1.17E-10 | 5.54E-09 | Respiratory burst oxidase homolog protein C |
| Unigene3581_All | 9.94 | 20.29 | 1.03 | up | 2.51E-05 | 0.000622 | NADPH oxidase |
| Unigene23385_All | 13.75 | 42.06 | 1.61 | up | 2.39E-12 | 1.32E-10 | Peroxidase |
| Unigene33444_All | 15.41 | 43.97 | 1.51 | up | 5.25E-06 | 0.000146 | Peroxidase |
| Unigene36593_All | 34.08 | 89.51 | 1.39 | up | 2.65E-09 | 1.11E-07 | Ascorbate oxidase |
| Unigene24091_All | 5.41 | 13.52 | 1.32 | up | 1.23E-08 | 4.81E-07 | L-ascorbate oxidase homolog |
| Unigene53679_All | 0.01 | 7.58 | 9.57 | up | 1.26E-06 | 3.84E-05 | Polyphenol oxidase (chloroplast) |
| Unigene24795_All | 2.74 | 18.54 | 2.76 | up | 2.09E-05 | 0.000528 | Polyphenol oxidase (chloroplast) |
| Unigene40171_All | 5.30 | 25.90 | 2.29 | up | 4.53E-11 | 2.25E-09 | Polyphenol oxidase (chloroplast) |
