# Supplementary material for: Gene expression profiles responses to aphid feeding in chrysanthemum (Chrysanthemum morifolium)
Source: BMC Genomics. 2014 Dec 2;15(1):1050. doi: 10.1186/1471-2164-15-1050 (PMC4265409; doi:10.1186/1471-2164-15-1050)
Supplement: Supplementary file 9 — Additional file 9: Table S8: Enzymes involved in reactive oxygen species (ROS) scavenging responding to aphid herbivory in the comparison between CK and Z (CK-VS-Z). The criteria used for assigning significance were: P-value < 0.05, FDR ≤ 0.001, and |log2Ratio(Z/CK)| ≥ 1. RPKM: reads per kb per million reads. CK: control; Z: mock puncture treatment. (DOC 37 KB) [file 12864_2014_6725_MOESM9_ESM.doc]

Additional file 9: Table S8. Enzymes involved in reactive oxygen species (ROS) scavenging responding to aphid herbivory in the comparison between CK and Z (CK-VS-Z). The criteria used for assigning significance were: *P*-value < 0.05, FDR ≤ 0.001, and |log_2_Ratio(Z/CK)| ≥ 1. RPKM: reads per kb per million reads. CK: control; Z: mock puncture treatment.

| GeneID | CK-RPKM | Z-RPKM | log_2_Ratio(Z/CK) | Up-Down-  Regulation(Z/CK) | P-value | FDR | Gene description |
| --- | --- | --- | --- | --- | --- | --- | --- |
| Unigene23385_All | 13.75 | 31.81 | 1.21 | up | 8.39E-07 | 4.29E-05 | Peroxidase |
| Unigene24091_All | 5.41 | 13.69 | 1.34 | up | 5.62E-09 | 3.75E-07 | L-ascorbate oxidase homolog |
| Unigene53679_All | 0.01 | 6.21 | 9.28 | up | 1.36E-05 | 0.000564 | Polyphenol oxidase (chloroplast) |
| Unigene24795_All | 2.74 | 22.88 | 3.06 | up | 4.95E-07 | 2.59E-05 | Polyphenol oxidase (chloroplast) |
| Unigene40171_All | 5.30 | 29.55 | 2.48 | up | 1.16E-13 | 1.18E-11 | Polyphenol oxidase (chloroplast) |
| Unigene9160_All | 10.57 | 25.87 | 1.29 | up | 4.87E-07 | 2.55E-05 | Polyphenol oxidase (chloroplast) |
