# Supplementary material for: Gene expression profiles responses to aphid feeding in chrysanthemum (Chrysanthemum morifolium)
Source: BMC Genomics. 2014 Dec 2;15(1):1050. doi: 10.1186/1471-2164-15-1050 (PMC4265409; doi:10.1186/1471-2164-15-1050)
Supplement: Supplementary file 10 — Additional file 10: Table S9: Differentially expressed genes (DEGs) involved in cell wall biosynthesis responding to aphid herbivory in the comparison between CK and Y (CK-VS-Y). The criteria used for assigning significance were: P-value < 0.05, FDR ≤ 0.001, and |log2Ratio(Y/CK)| ≥ 1. RPKM: reads per kb per million reads. CK: control; Y: aphid infestation treatment. (DOC 34 KB) [file 12864_2014_6725_MOESM10_ESM.doc]

Additional file 10: Table S9. Differentially expressed genes (DEGs) involved in cell wall biosynthesis responding to aphid herbivory in the comparison between CK and Y (CK-VS-Y). The criteria used for assigning significance were: *P*-value < 0.05, FDR ≤ 0.001, and |log_2_Ratio(Y/CK)| ≥ 1. RPKM: reads per kb per million reads. CK: control; Y: aphid infestation treatment.

| GeneID | CK-RPKM | Y-RPKM | log_2_Ratio(Y/CK) | Up-Down-  Regulation(Y/CK) | P-value | FDR | Gene description |
| --- | --- | --- | --- | --- | --- | --- | --- |
| Unigene11326_All | 16.83 | 86.69 | 2.36 | up | 2.19E-94 | 1.05E-91 | COBRA-like protein |
| Unigene2724_All | 17.56 | 60.87 | 1.79 | up | 9.64E-17 | 7.04E-15 | COBRA-like protein |
| Unigene22759_All | 17.58 | 54.15 | 1.62 | up | 6.26E-07 | 2.00E-05 | COBRA-like protein |
| Unigene25922_All | 6.84 | 24.43 | 1.84 | up | 2.21E-08 | 8.44E-07 | cellulose synthase-like protein D3-like |
| Unigene6200_All | 3.68 | 19.77 | 2.43 | up | 5.72E-15 | 3.74E-13 | cellulose synthase-like protein D3-like |
