# Supplementary material for: Gene expression profiles responses to aphid feeding in chrysanthemum (Chrysanthemum morifolium)
Source: BMC Genomics. 2014 Dec 2;15(1):1050. doi: 10.1186/1471-2164-15-1050 (PMC4265409; doi:10.1186/1471-2164-15-1050)
Supplement: Supplementary file 11 — Additional file 11: Table S10: Differentially expressed genes (DEGs) involved in cell wall biosynthesis responding to aphid herbivory in the comparison between CK and Z (CK-VS-Z). The criteria used for assigning significance were: P-value < 0.05, FDR ≤ 0.001, and |log2Ratio(Z/CK)| ≥ 1. RPKM: reads per kb per million reads. CK: control; Z: mock puncture treatment. (DOC 34 KB) [file 12864_2014_6725_MOESM11_ESM.doc]

Additional file 11: Table S10. Differentially expressed genes (DEGs) involved in cell wall biosynthesis responding to aphid herbivory in the comparison between CK and Z (CK-VS-Z). The criteria used for assigning significance were: *P*-value < 0.05, FDR ≤ 0.001, and |log_2_Ratio(Z/CK)| ≥ 1. RPKM: reads per kb per million reads. CK: control; Z: mock puncture treatment.

| GeneID | CK-RPKM | Z-RPKM | log_2_Ratio(Z/CK) | Up-Down-  Regulation(Z/CK) | P-value | FDR | Gene description |
| --- | --- | --- | --- | --- | --- | --- | --- |
| Unigene11326_All | 16.83 | 35.92 | 1.09 | up | 1.83E-14 | 1.99E-12 | COBRA-like protein |
| Unigene2724_All | 17.56 | 64.26 | 1.87 | up | 9.24E-19 | 1.31E-16 | COBRA-like protein |
| Unigene3108_All | 3.94 | 21.63 | 2.45 | up | 4.38E-14 | 4.60E-12 | Cellulose synthase-like A1 |
| Unigene25922_All | 6.84 | 29.70 | 2.12 | up | 8.93E-12 | 7.87E-10 | Cellulose synthase-like protein D3-like |
| Unigene6200_All | 3.68 | 15.11 | 2.04 | up | 7.80E-10 | 5.66E-08 | Cellulose synthase-like protein D3-like |
