# Supplementary material for: Gene expression profiles responses to aphid feeding in chrysanthemum (Chrysanthemum morifolium)
Source: BMC Genomics. 2014 Dec 2;15(1):1050. doi: 10.1186/1471-2164-15-1050 (PMC4265409; doi:10.1186/1471-2164-15-1050)
Supplement: Supplementary file 12 — Additional file 12: Table S11: Differentially expressed genes (DEGs) involved in secondary metabolites responding to aphid herbivory in the comparison between CK and Y (CK-VS-Y). The criteria used for assigning significance were: P-value < 0.05, FDR ≤ 0.001, and |log2Ratio(Y/CK)| ≥ 1. RPKM: reads per kb per million reads. CK: control; Y: aphid infestation treatment. (DOC 40 KB) [file 12864_2014_6725_MOESM12_ESM.doc]

Additional file 12: Table S11. Differentially expressed genes (DEGs) involved in secondary metabolites responding to aphid herbivory in the comparison between CK and Y (CK-VS-Y). The criteria used for assigning significance were: *P*-value < 0.05, FDR ≤ 0.001, and |log_2_Ratio(Y/CK)| ≥ 1. RPKM: reads per kb per million reads. CK: control; Y: aphid infestation treatment.

| GeneID | CK-RPKM | Y-RPKM | log_2_Ratio(Y/CK) | Up-Down-  Regulation(Y/CK) | P-value | FDR | Gene description |
| --- | --- | --- | --- | --- | --- | --- | --- |
| Unigene3981_All | 15.47 | 53.13 | 1.78 | up | 6.54E-15 | 4.23E-13 | phenylalanine ammonia-lyase |
| Unigene13361_All | 40.73 | 91.81 | 1.17 | up | 2.45E-07 | 8.27E-06 | phenylalanine ammonia-lyase |
| Unigene6380_All | 47.21 | 122.36 | 1.37 | up | 4.95E-64 | 1.51E-61 | phenylalanine ammonia-lyase |
| Unigene26066_All | 60.91 | 19.61 | -1.64 | down | 1.87E-47 | 3.96E-45 | flavonoid 3'-hydroxylase cytochrome P450 |
| Unigene29520_All | 23.45 | 79.91 | 1.77 | up | 3.23E-19 | 2.80E-17 | flavonoid 3' hydroxylase |
| Unigene29928_All | 6.01 | 29.03 | 2.27 | up | 7.05E-08 | 2.53E-06 | isoflavone reductase homolog A622-like |
| Unigene28528_All | 10.03 | 35.76 | 1.83 | up | 1.12E-07 | 3.91E-06 | isoflavone 2'-hydroxylase-like |
| Unigene3919_All | 2.05 | 14.41 | 2.81 | up | 1.16E-05 | 0.000308 | Terpene synthase |
