# Supplementary material for: Gene expression profiles responses to aphid feeding in chrysanthemum (Chrysanthemum morifolium)
Source: BMC Genomics. 2014 Dec 2;15(1):1050. doi: 10.1186/1471-2164-15-1050 (PMC4265409; doi:10.1186/1471-2164-15-1050)
Supplement: Supplementary file 13 — Additional file 13: Table S12: Differentially expressed genes (DEGs) involved in secondary metabolites responding to aphid herbivory in the comparison between CK and Z (CK-VS-Z). The criteria used for assigning significance were: P-value < 0.05, FDR ≤ 0.001, and |log2Ratio(Z/CK)| ≥ 1. RPKM: reads per kb per million reads. CK: control; Z: mock puncture treatment. (DOC 36 KB) [file 12864_2014_6725_MOESM13_ESM.doc]

Additional file 13: Table S12. Differentially expressed genes (DEGs) involved in secondary metabolites responding to aphid herbivory in the comparison between CK and Z (CK-VS-Z). The criteria used for assigning significance were: *P*-value < 0.05, FDR ≤ 0.001, and |log_2_Ratio(Z/CK)| ≥ 1. RPKM: reads per kb per million reads. CK: control; Z: mock puncture treatment.

| GeneID | CK-RPKM | Z-RPKM | log_2_Ratio(Z/CK) | Up-Down-  Regulation(Z/CK) | P-value | FDR | Gene description |
| --- | --- | --- | --- | --- | --- | --- | --- |
| Unigene3981_All | 15.47 | 66.28 | 2.10 | up | 1.38E-22 | 2.33E-20 | phenylalanine ammonia-lyase |
| Unigene6380_All | 47.21 | 127.16 | 1.43 | up | 2.65E-71 | 1.68E-68 | phenylalanine ammonia-lyase |
| Unigene13361_All | 40.73 | 89.28 | 1.13 | up | 6.43E-07 | 3.34E-05 | phenylalanine ammonia-lyase |
| Unigene26066_All | 60.91 | 15.82 | -1.94 | down | 7.49E-61 | 4.07E-58 | flavonoid 3'-hydroxylase cytochrome P450 |
| Unigene29520_All | 23.45 | 74.89 | 1.68 | up | 4.69E-17 | 6.04E-15 | flavonoid 3' hydroxylase |
| Unigene26695_All | 4.75 | 12.40 | 1.38 | up | 1.30E-05 | 0.00054 | Terpene synthase |
