# Supplementary material for: Gene expression profiles responses to aphid feeding in chrysanthemum (Chrysanthemum morifolium)
Source: BMC Genomics. 2014 Dec 2;15(1):1050. doi: 10.1186/1471-2164-15-1050 (PMC4265409; doi:10.1186/1471-2164-15-1050)
Supplement: Supplementary file 14 — Additional file 14: Table S13.: Differentially expressed photosynthesis-related genes responding to aphid herbivory in the comparison between CK and Y (CK-VS-Y). The criteria used for assigning significance were: P-value < 0.05, FDR ≤ 0.001, and |log2Ratio(Y/CK)| ≥ 1. RPKM: reads per kb per million reads. CK: control; Y: aphid infestation treatment. (DOC 29 KB) [file 12864_2014_6725_MOESM14_ESM.doc]

Additional file 14: Table S13. Differentially expressed photosynthesis-related genes responding to aphid herbivory in the comparison between CK and Y (CK-VS-Y). The criteria used for assigning significance were: *P*-value < 0.05, FDR ≤ 0.001, and |log_2_Ratio(Y/CK)| ≥ 1. RPKM: reads per kb per million reads. CK: control; Y: aphid infestation treatment.

| GeneID | CK-RPKM | Y-RPKM | log_2_Ratio(Y/CK) | Up-Down-  Regulation(Y/CK) | P-value | FDR | Gene description |
| --- | --- | --- | --- | --- | --- | --- | --- |
| Unigene24131_All | 9.24 | 19.98 | 1.11 | up | 8.91E-08 | 3.15E-06 | Photosystem II cytochrome b559 subunit alpha |
| Unigene9460_All | 12.34 | 31.40 | 1.35 | up | 9.13E-36 | 1.43E-33 | Photosystem I P700 apoprotein A2 |
