# Supplementary material for: Gene expression profiles responses to aphid feeding in chrysanthemum (Chrysanthemum morifolium)
Source: BMC Genomics. 2014 Dec 2;15(1):1050. doi: 10.1186/1471-2164-15-1050 (PMC4265409; doi:10.1186/1471-2164-15-1050)
Supplement: Supplementary file 15 — Additional file 15: Table S14: Differentially expressed nucleotide-binding site-leucine-rich repeat (NBS-LRR) genes responding to aphid herbivory in the comparison between CK and Y (CK-VS-Y). The criteria used for assigning significance were: P-value < 0.05, FDR ≤ 0.001, and |log2Ratio(Y/CK)| ≥ 1. RPKM: reads per kb per million reads. CK: control; Y: aphid infestation treatment. (DOC 28 KB) [file 12864_2014_6725_MOESM15_ESM.doc]

Additional file 15: Table S14. Differentially expressed nucleotide-binding site-leucine-rich repeat (NBS-LRR) genes responding to aphid herbivory in the comparison between CK and Y (CK-VS-Y). The criteria used for assigning significance were: *P*-value < 0.05, FDR ≤ 0.001, and |log_2_Ratio(Y/CK)| ≥ 1. RPKM: reads per kb per million reads. CK: control; Y: aphid infestation treatment.

| GeneID | CK-RPKM | Y-RPKM | log_2_Ratio(Y/CK) | Up-Down-  Regulation(Y/CK) | P-value | FDR | Gene description |
| --- | --- | --- | --- | --- | --- | --- | --- |
| Unigene3633_All | 45.98 | 193.41 | 2.07 | up | 7.42E-87 | 3.24E-84 | NBS-LRR resistance protein |
| Unigene14351_All | 9.21 | 21.54 | 1.23 | up | 8.96E-08 | 3.17E-06 | TIR-NBS-LRR resistance-like protein RGC151 |
